# Absence of spermatozoal CD46 protein expression and associated rapid acrosome reaction rate in striped field mice (Apodemus agrarius)

**DOI:** 10.1186/1477-7827-7-29

**Published:** 2009-04-16

**Authors:** Leanne E Clift, Petra Andrlikova, Michaela Frolikova, Pavel Stopka, Josef Bryja, Brian F Flanagan, Peter M Johnson, Katerina Dvorakova-Hortova

**Affiliations:** 1 Division of Immunology, School of Infection and Host Defence, University of Liverpool, Liverpool, UK; 2 Department of Zoology, Faculty of Science, Charles University, Prague, Czech Republic; 3 Department of Population Biology, Institute of Vertebrate Biology, Academy of Sciences of the Czech Republic, Brno, Czech Republic; 4 Department of Cell Biology, Faculty of Science, Charles University, Prague, Czech Republic

## Abstract

**Background:**

In rodents, the cell surface complement regulatory protein CD46 is expressed solely on the spermatozoal acrosome membrane. Ablation of the CD46 gene is associated with a faster acrosome reaction. Sperm from Apodemus flavicollis (yellow-necked field mice), A. microps (pygmy field mice) and A. sylvaticus (European wood mice) fail to express CD46 protein and exhibit a more rapid acrosome reaction rate than Mus (house mice) or BALB/c mice. A. agrarius (striped field mice) belong to a different Apodemus subgenus and have pronounced promiscuity and large relative testis size. The aim of this study was to determine whether A. agrarius sperm fail to express CD46 protein and, if so, whether A. agrarius have a faster acrosome reaction than Mus.

**Methods:**

Reverse transcription polymerase chain reaction (RT-PCR) was used to assess whether A. agrarius transcribe testicular CD46 mRNA. RT-PCR was supplemented with 3'- and 5'-rapid amplification of cDNA ends to determine the complete nucleotide sequence of A. agrarius CD46. Fluorescence microscopy was used to assess whether CD46 protein is expressed by A. agrarius sperm. The acrosome status of A. agrarius sperm was calculated over time by immunocytochemistry using peanut agglutinin lectin.

**Results:**

We demonstrate that A. agrarius mice transcribe two unique alternatively spliced testicular CD46 mRNA transcripts, both lacking exon 7, which differ from those described previously in other Apodemus species. The larger A. agrarius CD46 transcript has an insert between exons 10 and 11 which, if translated, would result in a novel cytoplasmic tail. In addition, A. agrarius CD46 transcripts have an extended AU-rich 3'-untranslated region (UTR) and a truncated 5'-UTR, resulting in failure to express spermatozoal CD46 protein. We show that A. agrarius has a significantly faster spontaneous acrosome reaction rate than A. sylvaticus and Mus.

**Conclusion:**

Absence of CD46 protein expression is associated with acrosomal instability in rodents. A. agrarius mice express novel CD46 transcripts, resulting in the trade of spermatozoal CD46 protein expression for a rapid acrosome reaction rate, in common with other species of field mice. This provides a strategy to increase competitive sperm advantage for individuals, leading to faster fertilisation in this highly promiscuous genus.

## Background

There is pronounced sperm competition in species belonging to the *Apodemus *genus (field mice), which is reflected by their disproportionately large testes [1,2]. There is also an association between relative testis mass and the shape of the apical hook in the falciform head of murine sperm [3]. The extremely long apical hook of *A. sylvaticus *spermatozoa enables them to intertwine, forming in vivo trains of up to 100 cells [4]. These sperm formations have significantly increased velocity and thrusting force compared with an individual spermatozoon. The *A. sylvaticus *sperm trains must dissociate prior to fertilisation, which may be achieved by a proportion of sperm undergoing a spontaneous acrosome reaction [4].

CD46 (membrane cofactor protein) is a widely expressed cell surface complement regulatory (CReg) protein in humans. In contrast, in rodents, CD46 protein expression is not widespread but instead is restricted solely to the spermatozoal acrosomal membrane, suggesting that CD46 has a role in the reproductive process [5-9]. The murine CD46 gene contains exons encoding four short consensus repeat (SCR) regions, a serine/threonine/proline-rich (STP) region, a juxtamembranous region of unknown function (UK), a transmembrane (TM) region and a cytoplasmic tail (CYT) region [10,11].

Disruption of the CD46 gene in laboratory mice induced a faster acrosome reaction rate compared with wild-type control mice [5]. Sperm from wild-caught *A. flavicollis, A. microps *and *A. sylvaticus*, belonging to the *Sylvaemus *subgenus, have been shown to exhibit a more rapid acrosome reaction rate than wild-caught *Mus musculus *or inbred BALB/c laboratory mice [12]. Abnormal testicular CD46 mRNA transcripts lacking exons 5–7 and 6–7, together with extended 3'- and truncated 5'-UTRs, were detected in these species, resulting in failure to express CD46 protein on testicular or epididymal sperm. It was proposed that these species have traded CD46 protein expression for acrosomal instability to favour more rapid fertilisation [12].

In contrast to the aforementioned *Apodemus *species, *A. agrarius *mice belong to a nominotypical subgenus of *Apodemus *[13,14] and have the highest level of promiscuity in any rodent species studied to date [15,16]. As in the ecologically diverse *Apodemus *species studied previously, there is pronounced sperm competition in *A. agrarius *for individual mating success.

The aim of the present study was to determine whether sperm from wild-caught *A. agrarius *mice also fail to express CD46 protein and, if so, whether *A. agrarius *have a faster acrosome reaction than *Mus*. This would reveal whether failure to express spermatozoal CD46 protein is widespread in the *Apodemus *genus or confined to *Apodemus *species within particular geographic ranges.

We demonstrate that *A. agrarius *mice transcribe two unique alternatively spliced testicular CD46 mRNA transcripts, with extended 3'- and truncated 5'-UTRs, resulting in failure to express spermatozoal CD46 protein. We show also that *A. agrarius *has a significantly faster spontaneous acrosome reaction rate than both *A. sylvaticus *and *Mus*. This study provides further support in favour of a role for CD46 in stabilisation of the acrosomal membrane in rodents.

## Methods

### Mice

Male *A. agrarius *and *A. sylvaticus *mice were caught from Slovakia and the Czech Republic, respectively. *M. m. musculus *mice were caught from around Buskovice (Czech Republic). All mice had reached sexual maturity. Wild-caught *A. agrarius, A. sylvaticus *and *M. m. musculus*, as well as inbred BALB/c mice, were maintained in a pathogen-free facility at the Department of Zoology, Charles University, Prague, Czech Republic. All animal procedures were carried out in accordance with the Animal (Scientific Procedure) Regulations and subjected to review by the local Ethical Committee.

### Testicular RNA extraction and reverse transcription

Testicular RNA was extracted using ice-cold Trizol reagent (Invitrogen, Paisley, UK) according to the protocol provided. RNA was stored at -20°C in DNAse- and RNAse-free water. Reverse transcription (RT) of testicular RNA was performed using Superscript II reverse transcriptase (Invitrogen, Paisley, UK).

### RT-PCR

RT-PCR reactions were conducted according to the ReddyMix Taq ×2 protocol (ABgene, Epsom, UK). The primers used in RT-PCR reactions when amplicons were subsequently sequenced are shown in Table 1. A murine ADAM2 (fertilin-beta) primer set was used in positive control reactions. 10 μl of CD46 PCR products and 2 μl of ADAM2 PCR products were visualised by 2% agarose gel electrophoresis and purified using the standard QIAquick PCR purification kit gel extraction protocol (Qiagen, Crawley, UK). Testicular CD46 amplicons were sequenced by primer extension using an ABI PRISM Sequence Detection System at Lark Technologies (Takeley, UK).

**Table 1:** CD46 primers used for RT-PCR, 5'-RACE and 3'-RACE reactions where amplicons were subsequently sequenced.

	**Primer sequences**	**Primer descriptions**	**Species of origin**
RT-PCR	CCTTCTGTTTCTGCTGTCT	CD46 exon 1, sense	*Mm, As*
	AATCATACATGGGTCCCTA	CD46 exon 2, sense	*Mm, As*
	TAGGGACCCATGTATGATT	CD46 exon 2, anti-sense	*Mm, As*
	ACTACATAGATGGCAG	CD46 exon 3, sense	*Mm, As*
	CTGCCATCTATGTAGT	CD46 exon 3, anti-sense	*Mm, As*
	ATTGTTTACCACCTCCA	CD46 exon 5, sense	*Mm, As*
	TGGAACACACACCTTTAC	CD46 exon 5, sense	*As*
	CGATCAAAGCAACAATC	CD46 exon 9, anti-sense	*As*
	TTCATCTTGCTGCAGATAC	CD46 exon 11, anti-sense	*Mm, As*
			
3'-RACE	TGGAACACACACCTTTAC	Gene-specific primer 1 (GSP), CD46 exon 5	*As*
	AAATGTATCTGCAGCAAG	GSP2, CD46 exon 11	*Mm, As*
	GACTCGAGTCGACATCGA	Anchor primer (AP)	-
	GACTCGAGTCGACATCGA(T)_17_G	AP-polyT-G	-
			
5'-RACE	AATTTAGCTCGAGCACC	GSP3, CD46 exon 3	*Mm*
	CTGCCATCTATGTAGT	GSP4, CD46 exon 3	*Mm, As*
	TAGGGACCCATGTATGATT	GSP5, CD46 exon 2	*Mm, As*
	GACTCGAGTCGACATCGA	AP	-
	GACTCGAGTCGACATCGA(G)_17_	AP-polyG	-

Key: *Mus musculus musculus *(*Mm*); *Apodemus sylvaticus *(*As*).

### Rapid amplification of cDNA ends (RACE)

The 3'-RACE protocol was identical to the Invitrogen 3'-RACE kit protocol, except that different anchor primers were used (Table 1) and an additional nested PCR step was added. The 5'-RACE protocol was based on the Invitrogen 5'-RACE kit protocol, with the following modifications. Firstly, cDNA was not treated with RNAse and was ethanol precipitated. 16.5 μl of cDNA was used in the tailing step rather than 6.5 μl, and samples were incubated with terminal deoxynucleotide transferase for 30 minutes instead of 10 minutes. Also, different anchor primers were used (Table 1) and an additional nested PCR step was added.

### Antibodies

A rabbit anti-*A. sylvaticus *CD46 peptide polyclonal antiserum was raised against a 14-mer peptide (PFEAMELKGTPKLY) from the predicted SCR1 domain of *A. sylvaticus *CD46, as previously described [12]. This high-titre polyclonal antiserum was affinity-purified against non-conjugated peptide immobilised to an agarose gel support using a Sulfolink kit (Perbio Science, Cramlington, UK) and eluted with 100 mM glycine buffer (pH2.8). A rat anti-rat CD46 polyclonal antiserum generated using a recombinant fusion protein comprising IgG-Fc and rat SCR2-3 CD46 domains was a kind gift of B. P. Morgan, C. L. Harris and M. Mizuno, Cardiff University School of Medicine [17].

### Immunocytochemistry

Epididymal sperm smears were permeabilised for 10 minutes in acetone pre-cooled at -20°C, air-dried and blocked using 3% normal goat serum for 1 hour. Slides were incubated with primary antibodies for 1 hour, followed by secondary antibodies for 30 minutes at optimised dilutions in phosphate-buffered isotonic saline. Rabbit pre-immune serum was used as a negative control, whereas the monoclonal antibody 18.6, which recognises an acrosome-associated antigen [18], was used as a positive control and was a kind gift of H. D. M. Moore, University of Sheffield. Slides were mounted and DNA was stained using Vectashield mounting medium with DAPI (Vector Laboratories, Peterborough, UK). Slides were visualised using an epifluorescence microscope (Olympus, Prague, Czech Republic) and images taken using a TCS SP2 RS high-speed confocal/two-photon system (Leica, Prague, Czech Republic).

### Acrosome reaction rate measurement

The distal region of the tail of the epididymis was placed into PBS for 10 minutes at 37°C under 5% CO_2_. The spermatozoa recovered were capacitated at a concentration of 5 × 10^6 ^sperm/ml in M2 fertilisation medium (Sigma-Aldrich, Prague, Czech Republic) under paraffin oil. Spermatozoa were collected at 5, 10, 20, 40, 80 and 120 minutes, stained to assess acrosomal status using 5 μM Alexa peanut agglutinin (PNA) lectin (Molecular Probes, Prague, Czech Republic) and immediately assessed under an epifluorescent microscope (Olympus, Prague, Czech Republic). Spermatozoa motility and viability were examined throughout experiments with a Sperm Viability Kit (Molecular Probes, Prague, Czech Republic) using an inverted microscope with a stage thermostatically controlled at 37°C. Spermatozoa viability was always greater than 86%.

## Results

### *A. agrarius *mice express two unique alternatively spliced testicular CD46 transcripts

**Figure 1:**
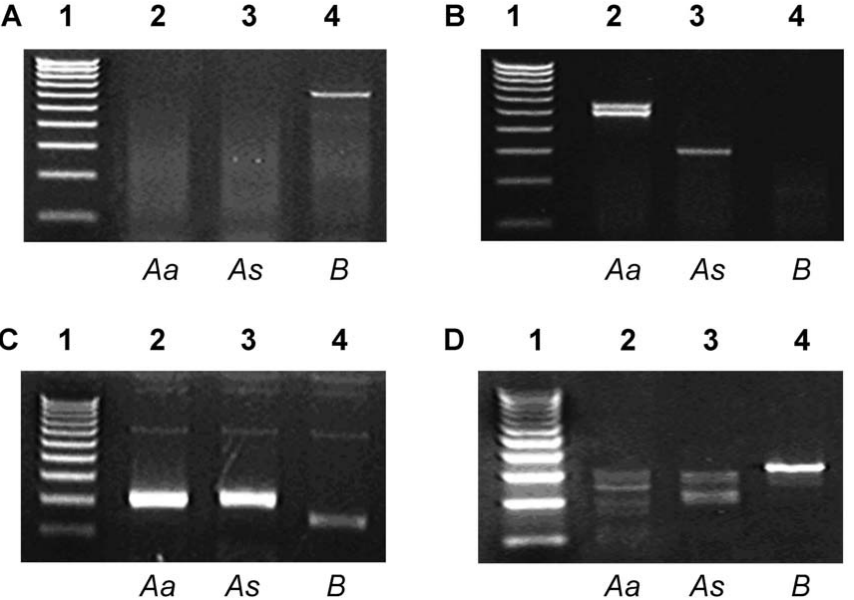
***A. agrarius *express two novel testicular CD46 transcripts with extended 3'- and truncated 5'-untranslated regions**. (**A**) Lane 1, 1000 nt ladder. Lanes 2–4, CD46 exon 5–11 primer set giving no product from *A. agrarius *or *A. sylvaticus *testicular RNA, but a 585 bp product from BALB/c testicular RNA. (**B**) Lane 1, 1000 nt ladder. Lanes 2–4, CD46 exon 5–11 primer set using an *A. sylvaticus*-specific exon 5 primer giving two products of 509 bp and 564 bp from *A. agrarius *testicular RNA, and a single 326 bp product from *A. sylvaticus *testicular RNA, but no product from BALB/c testicular RNA. **(C)** Lane 1, 1000 nt ladder. Lanes 2–4, 3'-rapid amplification of cDNA ends (3'-RACE) reactions giving products of 204 bp from *A. agrarius* and *A. sylvaticus* testicular RNA, and a product of 150 bp from BALB/c testicular RNA. **(D) **Lane 1, 1000 nt ladder. Lanes 2–4, 5'-RACE reactions giving a product of 353 bp from BALB/c testicular RNA, and various truncated products from *A. agrarius *and *A. sylvaticus *testicular RNA. Key: Aa, A. agrarius; As, A. sylvaticus; B, BALB/c.

RT-PCR was conducted using testicular mRNA derived from two wild-caught *A. agrarius *mice to ascertain whether *A. agrarius *express unusual CD46 transcripts. Use of a BALB/c-specific exon 5–11 primer set (Table 1) generated no product from *A. agrarius *or *A. sylvaticus *testicular mRNA (Figure 1A), whereas use of an *A. sylvaticus-*specific exon 5–11 primer set (Table 1) generated two cDNA amplicons from *A. agrarius *testicular mRNA, which were both larger (509 and 564 bp) than the amplicon generated from *A. sylvaticus *testicular mRNA (326 bp) (Figure 1B).

**Figure 2:**
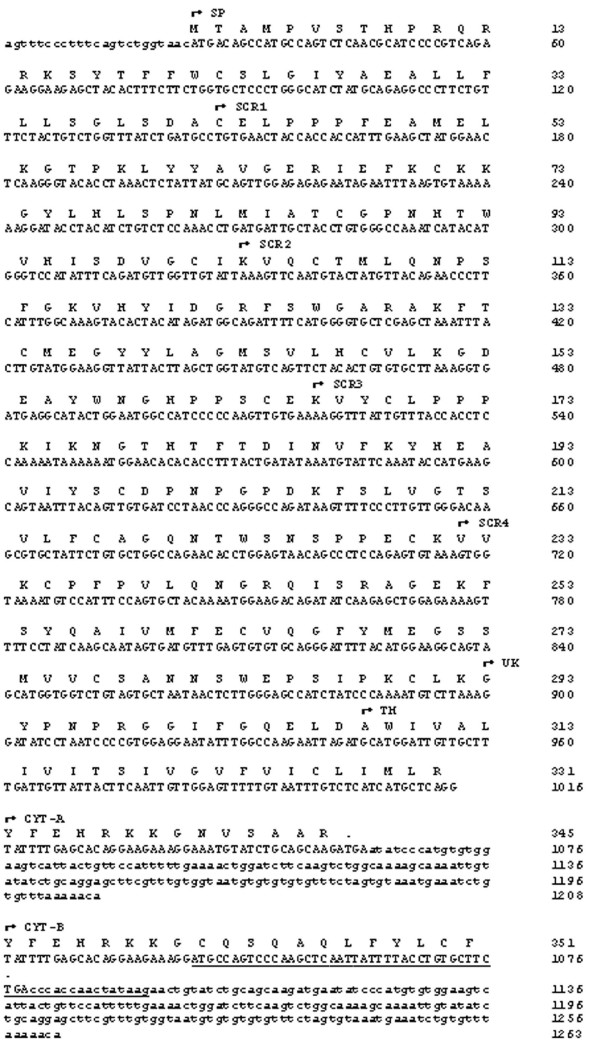
**Complete nucleotide and predicted amino acid sequence of *A. agrarius *testicular CD46**. The nucleotide sequences of the signal peptide (SP) to transmembrane (TM) domains are identical in both *A. agrarius *CD46 isoforms. Both isoforms are missing the serine/threonine/proline-rich (STP) domain. However, the *A. agrarius *CD46 isoforms have alternative cytoplasmic tails (CYT-A and CYT-B). The larger isoform has a 55 bp insertion (underlined) between exons 10 and 11, resulting in a longer cytoplasmic tail (CYT-B). *A. agrarius *CD46 mRNA sequences are available on the GenBank database [GenBank: FJ211179] and [GenBank: FJ211180].

*A. agrarius *CD46 amplicons were purified and sequenced. The smaller *A. agrarius *CD46 amplicon was 183 bp larger than the corresponding *A. sylvaticus *amplicon. Sequencing revealed that the disparity in size was due to the absence only of exon 7 from the larger *A. agrarius *transcript compared to the absence of both exons 6 and 7 from the *A. sylvaticus *CD46 transcript. The complete nucleotide and predicted amino acid sequence of the smaller *A. agrarius *CD46 isoform is shown in Figure 2.

Sequencing of the larger *A. agrarius *exon 5–11 amplicon revealed that exon 7 is also absent from this transcript. However, there was an additional 55 nucleotides inserted between exons 10 and 11. This 55 nucleotide insert had 96% homology to a 55 nucleotide sequence within intron 10 of the C57/BL mouse CD46 gene. Therefore, this 55 nucleotide insert in the larger *A. agrarius *CD46 transcript is likely to have been encoded from the equivalent intronic region of the *A. agrarius *CD46 gene. Figure 2 shows the location and nucleotide sequence of this 55 nucleotide insertion.

Two nucleotide polymorphisms were revealed in *A. agrarius *CD46 mRNA, at nucleotides 6 (C or T) and 125 (A or G) (Figure 2). This is consistent with the previous observation that both inter- and intra-species polymorphisms in the CD46 gene occur in the *Apodemus *genus, unlike in the *Mus *genus [12]. Surprisingly, none of the alternatively spliced CD46 transcripts previously identified in other species of the *Apodemus *genus (Johnson *et al*., 2007) were detected in the testis of *A. agrarius*. Also, no CD46 transcripts incorporating all exons were detected using *A. agrarius *testicular RNA.

### *A. agrarius *testicular CD46 transcripts have an extended 3'- and truncated 5'-untranslated region

RT-PCR was supplemented with 3'- and 5'-RACE to obtain the complete nucleotide sequence of *A. agrarius *CD46 mRNA. Like CD46 transcripts from other species of the *Apodemus *genus, testicular CD46 transcripts from *A. agrarius *have a 54 nucleotide AU-rich (70.4%) extension to the 3'-UTR (Figure 1C). In addition, the majority of *A. agrarius *5'-RACE amplicons terminated prematurely within exons 1 and 2 (Figure 1D).

### CD46 protein is not expressed by *A. agrarius *epididymal sperm

Amino acid sequences were predicted from the *A. agrarius *CD46 mRNA sequence data (Figure 2). If translated *in vivo*, the *A. agrarius *CD46 proteins would be lacking the STP domain. The exon 7 splicing event did not disrupt the open reading frame, and hence could not introduce a premature termination codon. However, the 55 nucleotide insertion between exons 10 and 11 of the larger *A. agrarius *CD46 transcript introduced a shift in the open reading frame, resulting in an alternative termination codon (Figure 2). Consequently, if translated, the larger *A. agrarius *CD46 isoform would have a slightly longer cytoplasmic tail (Figure 2, CYT-B) with a different amino acid sequence to the smaller isoform (Figure 2, CYT-A). The predicted *A. agrarius *CD46 proteins differ from those predicted in other *Apodemus *species, which would lack the SCR3, SCR4 and STP domains [12].

**Figure 3:**
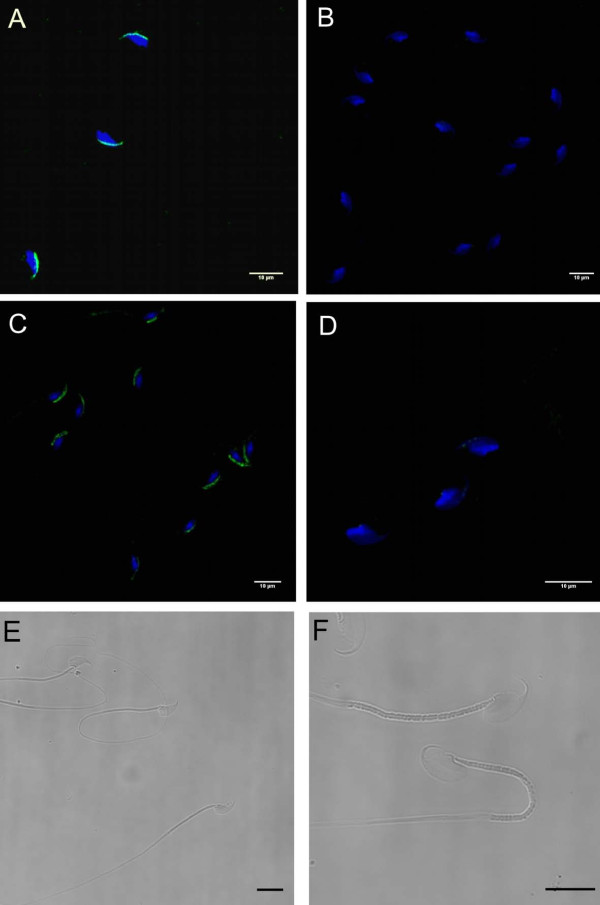
***A. agrarius *epididymal spermatozoa fail to express CD46 protein**. Immunostaining for CD46 (green) in the acrosomal region of epididymal sperm from *M. m. musculus *(**A **and **C**) and *A. agrarius *(**B **and **D**) using an affinity-purified rabbit polyclonal antibody against *A. sylvaticus *CD46 SCR1 peptide (**A **and **B**) or a rat polyclonal antiserum against rat CD46 (**C **and **D**). Phase contrast micrographs showing the head morphology of *M. m. musculus *(**E**) and *A. agrarius *(**F**) sperm. Nuclei are counterstained with DAPI (blue). Scale bars represent 10 μm.

Immunocytochemistry was conducted using sperm from wild-caught *A. agrarius *mice to determine whether CD46 protein is indeed expressed. No staining was observed on the acrosomal membrane of *A. agrarius *epididymal sperm using an affinity-purified rabbit anti-*A. sylvaticus *CD46 peptide polyclonal antibody or a rat anti-rat CD46 polyclonal antiserum; in contrast, strong acrosomal staining was observed on *M. m. musculus *epididymal sperm using both antibodies (Figure 3).

### *A. agrarius *mice have an accelerated acrosome reaction rate

**Figure 4:**
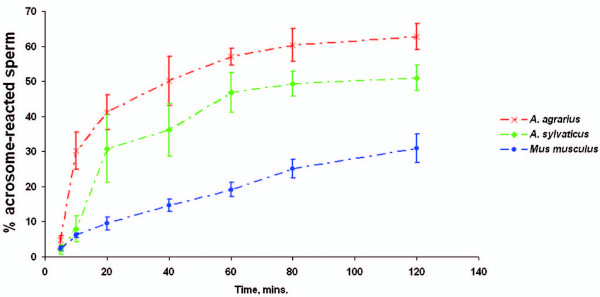
***A. agrarius *spermatozoa have an accelerated spontaneous acrosome reaction rate**. The spontaneous acrosome reaction rate is significantly faster for *A. agrarius *sperm compared with *A. sylvaticus *(p < 0.001) and *M. m. musculus *(p < 0.001) sperm. Each data point represents the average acrosome reaction rate of eight mice.

The spontaneous acrosome reaction rate of epididymal sperm was measured and compared with wild-caught *A. agrarius*, *A. sylvaticus *and *M. m. musculus *(Figure 4). The Post-hoc Tukey test demonstrated that, consistent with other species of the *Apodemus *genus, sperm from *A. agrarius *mice have a significantly accelerated acrosome reaction rate compared to sperm from wild-caught *M. m. musculus *(p < 0.001). The acrosome reaction rate of *A. agrarius *sperm was also significantly faster than that of *A. sylvaticus *sperm (p < 0.001). These findings add strength to the hypothesis that failure of sperm to express CD46 protein destabilises the acrosome, resulting in an accelerated acrosome reaction rate.

## Discussion

Wild-caught *A. agrarius *mice transcribe two unique alternatively spliced testicular CD46 mRNA transcripts which are both different from those described previously in other *Apodemus *species [12]. Exon 7, encoding the STP domain, has been spliced from both of the *A. agrarius *CD46 transcripts. The larger *A. agrarius *transcript has a 55 nucleotide insert between exons 10 and 11, encoded from intron 10 of the CD46 gene. This insertion disrupts the open reading frame, resulting in an alternative termination codon, located 33 nucleotides later than in the smaller CD46 transcript. If translated *in vivo*, the larger isoform would have an alternative novel 20 amino acid cytoplasmic tail.

The acrosome of wild-caught *M. m. musculus *epididymal sperm showed strong positive staining using both an affinity-purified rabbit anti-*A. sylvaticus *CD46 peptide polyclonal antibody and a rat anti-rat CD46 polyclonal antiserum. In contrast, no staining was observed on the acrosomal membrane of *A. agrarius *epididymal sperm. The predicted *A. sylvaticus *CD46 peptide, against which the polyclonal antibody was raised (Johnson et al. 2007), is identical to the corresponding amino acid sequence in *A. agrarius*. Therefore, if CD46 protein is indeed expressed by *A. agrarius *sperm, it should have been detected using this antibody.

As in the other species of the *Apodemus *genus studied previously [12], testicular CD46 transcripts in *A. agrarius *have a 54 nucleotide AU-rich extension to the 3'-UTR and a truncated 5'-UTR. Several mechanisms could explain the absence of CD46 protein expression by *A. agrarius *sperm. 3'- and 5'-UTRs can modulate the transport of mRNA out of the nucleus, translation efficacy, subcellular localisation and mRNA stability [19-21]. AU-rich elements (AREs) are sequence motifs located in the 3'-UTR of some mRNAs and are implicated in mRNA stability [22,23]. *In vitro *studies indicate that degradation of mRNAs containing AREs requires the decapping/5'-3' decay pathway [24]. The 3'-UTR of *A. agrarius *CD46 transcripts resembles a class III ARE. It is possible that the 'ARE' in the 3'-UTR of *A. agrarius *CD46 induces degradation of transcripts in the 5'-3' direction in a deadenylation-independent manner. 5'-UTRs contain several elements involved in translational control, including a 7-methyl-guanosine cap, an internal ribosome entry site, hairpin-like secondary structures and structural motifs that attract RNA-binding proteins [25]. The majority of *A. agrarius *CD46 5'RACE amplicons terminated prematurely within exons 1 and 2. Therefore, most *A. agrarius *CD46 transcripts lack the 5'-UTR regulatory elements, the initiation codon and parts of exons encoding the signal peptide and the SCR1 domain, cumulating in failure to translate CD46 protein.

As in *A. flavicollis, A. microps *and *A. sylvaticus *[12], minor intra-species nucleotide polymorphisms occurred in *A. agrarius *CD46 transcripts. Several genes expressed in mammalian spermatozoa are rapidly evolving. This may, in part, result from selection on males to achieve rapid fertilisation of ova under sperm competition [26,27]. Thus, sexual selection in *Apodemus *could have driven the rapid divergence. Alternatively, and more likely, the high level of both intra- and inter-species CD46 polymorphisms in the *Apodemus *genus compared to the *Mus *genus, which has an invariant CD46 gene, may indicate neutral drift and that the *Apodemus *CD46 gene has escaped strict genetic control and is non-functional. This is supported by the fact that CD46 protein is not expressed by *Apodemus *sperm.

**Figure 5:**
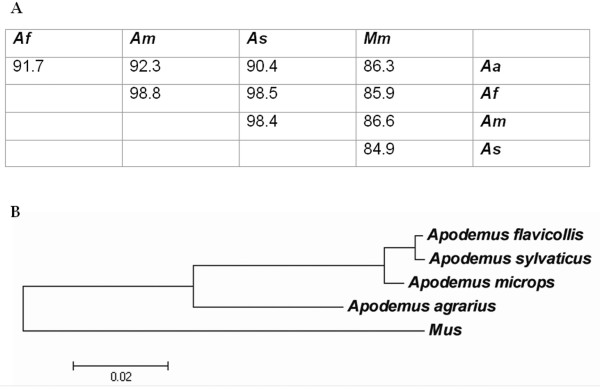
**Percentage homology and phylogenetic relationship between testicular CD46 transcripts from species of *Apodemus *and *Mus***. **(A) **The percentage homology of nucleotide sequences of testicular CD46 transcripts from species of the *Apodemus *and *Mus *genera. **(B) **The phylogenetic relationship (Neighbour-Joining tree) between the amino acid sequences of testicular CD46 from species of the *Apodemus *and *Mus *genera. The scale bar represents the distance between sequence pairs using the Poisson model with rate variation among sites. The accession numbers of *A. agrarius, A. flavicollis, A. microps, A. sylvaticus *and BALB/c (*Mus*) CD46 mRNA are [GenBank: FJ211179], [GenBank: EF216472], [GenBank: EF216473], [GenBank: EF216471] and [GenBank: NM_010778], respectively. Key: *A. agrarius *(*Aa*); *A. flavicollis *(*Af*); *A. microps *(*Am*); *A. sylvaticus *(*As*); *M. m. domesticus *and *M. m. musculus *(*Mm*).

*A. agrarius *CD46 consensus nucleotide sequences were aligned against the corresponding consensus sequences from the *Apodemus *and *Mus *species previously studied [12]. The percentage homologies of CD46 mRNA consensus sequences between different species of the *Apodemus *genus ranged from 90.4% to 98.8%. *A. flavicollis *and *A. microps *CD46 transcripts had the greatest homology, whereas *A. agrarius *and *A. sylvaticus *CD46 transcripts had the least homology (Figure 5).

The predicted CD46 amino acid sequences were utilised to construct a schematic phylogenetic tree to estimate the relationship between CD46 from species of the *Apodemus *and *Mus *genera (Figure 5). The tree was constructed using the Neighbour-Joining method, with complete deletion of gaps, Poisson correction and different evolutionary rates with a gamma parameter of one, using Molecular Evolutionary Genetic Analysis (MEGA) version 4.0 [28]. Analysis of nucleotide sequence homologies and the amino acid phylogenetic tree illustrate that, following the separation of *Mus *and *Apodemus*, the divergence of *A. agrarius *precedes the more recent divergence of *A. microps, A. sylvaticus *and *A. flavicollis*.

Exon 7 is spliced out of CD46 transcripts from all *Apodemus *species studied, but is present in *Mus *CD46 transcripts. This indicates that the genetic changes that cause exon 7 to be spliced from *Apodemus *CD46 pre-mRNA occurred following the divergence of *Apodemus *from *Mus*. In contrast, exon 5 and 6 splicing events were observed in *A. flavicollis, A. microps *and *A. sylvaticus *[12] but were not observed in *A. agrarius*, indicating that the polymorphism(s) responsible occurred after *A. flavicollis, A. microps *and *A. sylvaticus *diverged from *A. agrarius*. The CD46 phylogenetic tree is consistent with previous phylogenetic trees constructed using data derived from sequencing of the cytochrome b and the interphotoreceptor retinoid-binding protein genes, also indicating that species of the *Sylvaemus *subgenus (i.e. *A. flavicollis, A. microps *and *A. sylvaticus*) tend to be closely related to one another [29].

Like sperm from wild-caught *A. flavicollis, A. microps *and *A. sylvaticus*, sperm from wild-caught *A. agrarius *fail to express CD46 protein and have a significantly faster spontaneous acrosome reaction rate compared to wild-caught *Mus *and inbred BALB/c mice [12]. Dissociation of *Apodemus *sperm trains may be assisted by a significant proportion of sperm undergoing an accelerated acrosome reaction [4]. The high concentration of hydrolytic enzymes released may bypass zona pellucida (ZP) binding, enabling an acrosome-reacted sperm to fertilise the oocyte. Acrosome-reacted mouse sperm are able to fertilise ZP-free eggs and produce normal offspring [30]. Thus, spermatozoal CD46 protein expression may have been traded for an unstable acrosome, enabling the dissociation of sperm trains via a premature acrosome reaction and favoring more rapid fertilisation.

Interestingly, the acrosome reaction rate of *A. agrarius *sperm was also significantly faster than that of *A. sylvaticus *sperm. Sperm with the fastest binding rates are selected when multiple males are competing to bind with the oocyte [26]. *A. agrarius *mice have significantly larger testes and a slightly higher proportion of litters with multiple paternity than *A. sylvaticus *mice [16]. This suggests that *A. agrarius *sperm are subject to higher levels of sperm competition than *A. sylvaticus *sperm, which may have driven the selection of a faster acrosome reaction rate.

Sperm from CD46 gene-disrupted mice also have an accelerated spontaneous acrosome reaction rate compared to wild-type control mice [5], and *Apodemus *mice appear to be naturally occurring null phenotypic analogues of CD46^-/- ^knockout mice. Like CD46^-/- ^mice, *Apodemus *mice do not exhibit any abnormalities in spermatogenesis or fertilisation, supporting the concept that the primary role of CD46 on acrosome-reacted sperm is not protection against complement-mediated damage. Instead, spermatozoal CD46 in rodents may have an important role in maintaining acrosome integrity. The precise molecular mechanism by which this is achieved can only be speculated at present.

CD46 is a receptor for pathogenic *Neisseria *[31]. Levels of cytoplasmic calcium are elevated following binding of *Neisseria *pili to host cells, due to release of calcium from intracellular stores. Antibodies against CD46 block this increase in cytoplasmic calcium levels, indicating that CD46 is involved in signal transduction [32]. The induction of calcium mobilisation by CD46 signaling may be relevant to the acrosome reaction, and perhaps perturbed in mice that do not express CD46 protein or in promiscuous mice susceptible to venereal infection.

Disruption of spermatozoal CD46 could also affect the distribution of membrane proteins which associate with it, including β1 integrins [33]. Integrins interact with several cytoskeletal proteins, including actin [34]. CD46/CD3 costimulation of T cells results in morphological changes and actin relocalization [35]. Cytoskeleton restructuring throughout capacitation is crucial for successful membrane fusion prior to the acrosome reaction. CD46 associates with kinases (including Erk1/2 and MAPK) both directly and indirectly through integrins [35,36], and Erk1/2 and p38 MAPK are involved in the acrosome reaction [37].

CD46 may thus be involved in intracellular signaling events related to the acrosome reaction. Failure to express CD46 protein in *Apodemus*, including *A. agrarius*, may alter intracellular signaling and destabilise the acrosomal region.

## Conclusion

*A. agrarius *mice transcribe two unique alternatively spliced CD46 transcripts, with truncated 5'- and extended 3'-UTRs. The majority of *A. agrarius *CD46 transcripts are lacking 5'-UTR regulatory elements, in addition to the initiation codon, resulting in failure to translate CD46 protein. *A. agrarius *has a significantly faster acrosome reaction rate than *Mus *and, indeed, *A. sylvaticus*. Absence of CD46 protein expression is associated with acrosomal instability in rodents. Spermatozoal CD46 protein expression in *A. agrarius *may have been traded for an unstable acrosome, enabling the dissociation of sperm trains via a premature acrosome reaction. This favours more rapid fertilisation, providing an unexpected molecular mechanism to increase competitive sperm advantage in this highly promiscuous species.

## Competing interests

The authors declare that they have no competing interests.

## Authors' contributions

LEC carried out the molecular genetic studies and drafted the manuscript. PA and KDH conducted the acrosome reaction rate experiments. MF carried out immunocytochemistry experiments to determine CD46 expression. PS and JB carried out the phylogenetic analysis. PMJ and KDH conceived of the study, and participated in its design and coordination. PA, PS, JB, BFF, PMJ and KDH helped to revise manuscript drafts. All authors have approved the manuscript.

## References

[B1] Breed WG, Taylor J (2000). Body mass, testis mass and sperm size in murine rodents. J Mammal.

[B2] Bryja J, Stopka P (2005). Facultative promiscuity in a presumably monogamous mouse (*Apodemus microps*). Acta theriol.

[B3] Immler S, Moore HD, Breed WG, Birkhead TR (2007). By hook or by crook? Morphometry, competition and cooperation in rodent sperm. PLoS ONE.

[B4] Moore H, Dvorakova K, Jenkins N, Breed W (2002). Exceptional sperm cooperation in the wood mouse. Nature.

[B5] Inoue N, Ikawa M, Nakanishi T, Matsumoto M, Nomura M, Seya T, Okabe M (2003). Disruption of mouse CD46 causes an accelerated spontaneous acrosome reaction in sperm. Mol Cell Biol.

[B6] Mizuno M, Harris CL, Johnson PM, Morgan BP (2004). Rat membrane cofactor protein (MCP; CD46) is expressed only in the acrosome of developing and mature spermatozoa and mediates binding to immobilized activated C3. Biol Reprod.

[B7] Hosokawa M, Nonaka M, Okada N, Nonaka M, Okada H (1996). Molecular cloning of guinea pig membrane cofactor protein: preferential expression in testis. J Immunol.

[B8] Riley RC, Kemper C, Leung M, Atkinson JP (2002). Characterization of human membrane cofactor protein (MCP; CD46) on spermatozoa. Mol Reprod Dev.

[B9] Anderson DJ, Michaelson JS, Johnson PM (1989). Trophoblast/leukocyte-common antigen is expressed by human testicular germ cells and appears on the surface of acrosome-reacted sperm. Biol Reprod.

[B10] Miwa T, Nonaka M, Okada N, Wakana S, Shiroishi T, Okada H (1998). Molecular cloning of rat and mouse membrane cofactor protein (MCP, CD46): preferential expression in testis and close linkage between the mouse *Mcp *and *Cr2 *genes on distal chromosome 1. Immunogenetics.

[B11] Tsujimura A, Shida K, Kitamura M, Nomura M, Takeda J, Tanaka H, Matsumoto M, Matsumiya K, Okuyama A, Nishimune Y, Okabe M, Seya T (1998). Molecular cloning of a murine homologue of membrane cofactor protein (CD46): preferential expression in testicular germ cells. Biochem J.

[B12] Johnson PM, Clift LE, Andrlikova P, Jursova M, Flanagan BF, Cummerson JA, Stopka P, Dvorakova-Hortova K (2007). Rapid sperm acrosome reaction in the absence of acrosomal CD46 expression in promiscuous field mice (*Apodemus*). Reproduction.

[B13] Michaux JR, Chevret P, Filippucci MG, Macholan M (2002). Phylogeny of the genus *Apodemus *with a special emphasis on the subgenus *Sylvaemus *using the nuclear IRBP gene and two mitochondrial markers: cytochrome b and 12S rRNA. Mol Phylogenet Evol.

[B14] Filippucci MG, Macholan M, Michaux JR (2002). Genetic variation and evolution in the genus *Apodemus *(Muridae: Rodentia). Biol J Linnean Soc.

[B15] Wolff JO, Macdonald DW (2004). Promiscuous females protect their offspring. Trends Ecol Evol.

[B16] Bryja J, Patzenhauerova H, Albrecht T, Mosansky L, Stanko M, Stopka P (2008). Varying levels of female promiscuity in four *Apodemus *mice species. Behav Ecol Sociobiol.

[B17] Mizuno M, Harris CL, Morgan BP (2007). Immunization with autologous CD46 generates a strong autoantibody response in rats that targets spermatozoa. J Reprod Immunol.

[B18] Moore HD, Smith CA, Hartman TD, Bye AP (1987). Visualization and characterization of the acrosome reaction of human spermatozoa by immunolocalization with monoclonal antibody. Gamete Res.

[B19] Maquat LE, Carmichael GG (2001). Quality control of mRNA function. Cell.

[B20] Bashirullah A, Cooperstock RL, Lipshitz HD (1998). RNA localization in development. Annu Rev Biochem.

[B21] Velden AW van der, Thomas AA (1999). The role of the 5' untranslated region of an mRNA in translation regulation during development. Int J Biochem Cell Biol.

[B22] Chen CY, Shyu AB (1995). AU-rich elements: characterization and importance in mRNA degradation. Trends Biochem Sci.

[B23] Xu L, Rahimpour R, Ran L, Kong C, Biragyn A, Andrews J, Devries M, Wang JM, Kelvin DJ (1997). Regulation of CCR2 chemokine receptor mRNA stability. J Leukoc Biol.

[B24] Stoecklin G, Mayo T, Anderson P (2006). ARE-mRNA degradation requires the 5'-3' decay pathway. EMBO Rep.

[B25] Mignone F, Gissi C, Liuni S, Pesole G (2002). Untranslated regions of mRNAs. Genome Biol.

[B26] Levitan DR, Ferrell DL (2006). Selection on gamete recognition proteins depends on sex, density, and genotype frequency. Science.

[B27] Torgerson DG, Kulathinal RJ, Singh RS (2002). Mammalian sperm proteins are rapidly evolving: evidence of positive selection in functionally diverse genes. Mol Biol Evol.

[B28] Tamura K, Dudley J, Nei M, Kumar S (2007). MEGA4: Molecular Evolutionary Genetics Analysis (MEGA) software version 4.0. Mol Biol Evol.

[B29] Serizawa K, Suzuki H, Tsuchiya K (2000). A phylogenetic view on species radiation in *Apodemus *inferred from variation of nuclear and mitochondrial genes. Biochem Genet.

[B30] Naito K, Toyoda Y, Yanagimachi R (1992). Production of normal mice from oocytes fertilized and developed without zonae pellucidae. Hum Reprod.

[B31] Kallstrom H, Liszewski MK, Atkinson JP, Jonsson AB (1997). Membrane cofactor protein (MCP or CD46) is a cellular pilus receptor for pathogenic *Neisseria*. Mol Microbiol.

[B32] Kallstrom H, Islam MS, Berggren PO, Jonsson AB (1998). Cell signaling by the type IV pili of pathogenic *Neisseria*. J Biol Chem.

[B33] Lozahic S, Christiansen D, Manie S, Gerlier D, Billard M, Boucheix C, Rubinstein E (2000). CD46 (membrane cofactor protein) associates with multiple beta1 integrins and tetraspans. Eur J Immunol.

[B34] Flier A van der, Sonnenberg A (2001). Function and interactions of integrins. Cell Tissue Res.

[B35] Zaffran Y, Destaing O, Roux A, Ory S, Nheu T, Jurdic P, Rabourdin-Combe C, Astier AL (2001). CD46/CD3 costimulation induces morphological changes of human T cells and activation of Vav, Rac, and extracellular signal-regulated kinase mitogen-activated protein kinase. J Immunol.

[B36] Wong TC, Yant S, Harder BJ, Korte-Sarfaty J, Hirano A (1997). The cytoplasmic domains of complement regulatory protein CD46 interact with multiple kinases in macrophages. J Leukoc Biol.

[B37] Almog T, Lazar S, Reiss N, Etkovitz N, Milch E, Rahamim N, Dobkin-Bekman M, Rotem R, Kalina M, Ramon J, Raziel A, Brietbart H, Seger R, Naor Z (2008). Identification of extracellular signal-regulated kinase 1/2 and p38 MAPK as regulators of human sperm motility and acrosome reaction and as predictors of poor spermatozoan quality. J Biol Chem.

